# Cardioprotection afforded by exercise training prior to myocardial infarction is associated with autonomic function improvement

**DOI:** 10.1186/1471-2261-14-84

**Published:** 2014-07-14

**Authors:** Fernando Rodrigues, Daniele Jardim Feriani, Catarina Andrade Barboza, Marcos Elias Vergilino Abssamra, Leandro Yanase Rocha, Nicolle Martins Carrozi, Cristiano Mostarda, Diego Figueroa, Gabriel Inacio Honorato Souza, Kátia De Angelis, Maria Cláudia Irigoyen, Bruno Rodrigues

**Affiliations:** 1 Human Movement Laboratory, Sao Judas Tadeu University (USJT), São Paulo, SP, Brazil; 2 Federal University of Maranhao (UFMA), São Luis, MA, Brazil; 3 Hypertension Unit, Heart Institute (InCor), Medical School of University of Sao Paulo, São Paulo, SP, Brazil; 4 Translational Physiology Laboratory, Universidade Nove de Julho (UNINOVE), São Paulo, SP, Brazil

**Keywords:** Exercise training, Myocardial infarction, Cardiac function, Autonomic nervous system

## Abstract

**Background:**

It has been suggested that exercise training (ET) protects against the pathological remodeling and ventricular dysfunction induced by myocardial infarction (MI). However, it remains unclear whether the positive adjustments on baroreflex and cardiac autonomic modulations promoted by ET may afford a cardioprotective mechanism. The aim of this study was to evaluate the effects of aerobic ET, prior to MI, on cardiac remodeling and function, as well as on baroreflex sensitivity and autonomic modulation in rats.

**Methods:**

Male Wistar rats were divided into 4 groups: sedentary rats submitted to *Sham* surgery (C); trained rats submitted to *Sham* surgery (TC); sedentary rats submitted to MI (I), trained rats submitted to MI (TI). *Sham* and MI were performed after ET period. After surgeries, echocardiographic, hemodynamic and autonomic (baroreflex sensitivity, cardiovascular autonomic modulation) evaluations were conducted.

**Results:**

Prior ET prevented an additional decline in exercise capacity in TI group in comparison with I. MI area was not modified by previous ET. ET was able to increase the survival and prevent additional left ventricle dysfunction in TI rats. Although changes in hemodynamic evaluations were not observed, ET prevented the decrease of baroreflex sensitivity, and autonomic dysfunction in TI animals when compared with I animals. Importantly, cardiac improvement was associated with the prevention of cardiac autonomic impairment in studied groups.

**Conclusions:**

Prior ET was effective in changing aerobic capacity, left ventricular morphology and function in rats undergoing MI. Furthermore, these cardioprotective effects were associated with attenuated cardiac autonomic dysfunction observed in trained rats. Although these cause-effect relationships can only be inferred, rather than confirmed, our study suggests that positive adaptations of autonomic function by ET can play a vital role in preventing changes associated with cardiovascular disease, particularly in relation to MI.

## Background

Coronary artery disease, together with myocardial infarction (MI), is the most prevalent cardiovascular disease (CVD), and commonly progresses to heart failure in affected individuals [1]. It has long been known that cigarette smoking, hypertension, hypercholesterolemia, diabetes mellitus, obesity and physical inactivity are the main risk factors of MI [2], and prevention strategies have been designed. After MI, autonomic imbalance is usually followed by abnormalities in the cardiorespiratory reflex control i.e., impaired baroreflex sensitivity and function, increased activation of ergoreflex and chemoreflex [3-6].Thus, autonomic imbalance came to be seen as a key element in the pathophysiology of ventricular dysfunction and failure [7].

Metabolic, cardiovascular, autonomic and anti-inflammatory benefits of a physically active life-style have led many researchers to suggest exercise training (ET) as an important non-pharmacological tool in the prevention and treatment of CVD [8-10]. The effectiveness of ET as a powerful tool in the treatment of MI abnormalities has been widely reported in clinical and experimental settings [11-17].

On the other hand, a few studies have evaluated how ET undertaken prior to a MI affected aerobic capacity, cardiac function and morphometry, as well as mortality rate. Despite distinct experimental designs and ET regimens, the data found in the literature suggest that ET protects against the pathological remodeling and ventricular dysfunction induced by MI in rodents [18-23]. Mechanisms such as the formation of collateral vessels [24], elevation of heat shock proteins [25], increased myocardial expression of cyclooxygenase [26], antioxidant protection [21] as well as anti-inflammatory role of exercise [27] have been implicated as triggers of cardioprotection. However, it remains unclear whether the positive adjustments on baroreflex and cardiac autonomic modulations promoted by ET may afford a cardioprotective mechanism.

Therefore, the present study was undertaken to investigate 1) whether ET prior to MI prevents cardiac dysfunction and morphometric derangements; 2) whether ET prior to MI changes mortality rate; and 3) whether the potential cardiac benefits of ET could be associated with preserved baroreflex sensitivity and cardiac autonomic modulation.

## Methods

### Animals

Experiments were performed in adult male Wistar rats (275-300 g) from the Animal House of the São Judas Tadeu University, São Paulo, Brazil. Rats were fed standard laboratory chow and water ad libitum. The animals were housed in collective polycarbonate cages in a temperature-controlled room (22-23°C) and under 54-55% humidity with a 12-h dark–light cycle (light 07:00-19:00 h). The experimental protocol was approved by the institutional animal care and use committee of the São Judas Tadeu University (008/2013), and this investigation was conducted in accordance with the Principles of Laboratory Animal Care formulated by the National Institutes of Health (National Institutes of Health Publication No., 96-23, Revised 1996).

The rats were randomly assigned to four groups: sedentary control rats submitted to *Sham* surgery (C, n = 8); trained control rats submitted to *Sham* surgery (TC, n = 8); sedentary rats submitted to MI surgery (I, n = 12), trained rats submitted to MI surgery (TI, n = 10).

### Exercise training

Sedentary and trained rats were adapted to the treadmill (10 minutes per day; 0.3 km/h) for 5 days. All animals were submitted to a maximal treadmill exercise test to determine aerobic capacity and exercise training intensity at the beginning of the protocol (initial evaluation), after 4 weeks (to training intensity adjustments, data not show), after ET protocol (final evaluation), and 2 days after *Sham* or MI surgeries. Our group previously demonstrated that maximal treadmill exercise test can detect differences in aerobic performance; since that the maximal running speed achieved in the test presented a good correlation with the maximum oxygen consumption [28].

ET was performed on a motorized treadmill at low-moderate intensity (50%-70% maximal running speed) for 1 hour a day, 5 days a week for 8 weeks, with a gradual increase in speed from 0.3 to 1.2 km/h [13].

### Myocardial infarction induction

Anaesthetized rats (80 mg/kg ketamine and 12 mg/kg xylazine, i.p.) underwent surgical occlusion of the left coronary artery, which resulted in MI as described previously [16,17]. Briefly, after intubation, animals were positive-pressure ventilated with room air at 2.5 mL, 65 strokes/minute with a pressure-cycled rodent ventilator (Harvard Apparatus, Model 683, Holliston, MA, USA). For induction of MI, a 2-cm left lateral thoracotomy was performed in the third intercostal space, and the left anterior descending coronary artery was occluded with a nylon (6.0) suture at approximately 1 mm from its origin below the tip of the left atrium. The C and TC animals underwent the same procedures except that myocardial ischemia was not induced – *Sham* surgery. The chest was closed with a silk suture.

### Echocardiographic evaluation

One day after *Sham* or MI surgeries, echocardiographic evaluations were performed by a blinded observer, under the guidelines of the American Society of Echocardiography. Rats were anaesthetized (80 mg/kg ketamine and 12 mg/kg xylazine, i.p.), and images were obtained with a 10-14 mHz linear transducer in a SEQUOIA 512 (Acuson Corporation, MountainView, CA, USA) for measurements of parameters: left ventricular mass (LVmass); left ventricular end-diameter during diastole (LVDD); relative wall thickness (RWT); fractional shortening (FS); ejection fraction (EF); E wave A wave ratio (E/A); left ventricular isovolumetric relaxation time (IVRT); myocardial performance index (MPI), as described in detail elsewhere [16,17].

The MI area was delimited taking into account the movement of LV walls during initial and final echocardiographic evaluations by a blinded observer. MI was defined by echocardiography as any segmental wall motion abnormality such as hypokinesis, akinesis and dyskinesis, as described previously [14,16,17]. Our group previously showed strong correlations between the MI area assessed by echocardiogram and *post mortem* histological analysis [14,16,17,29], showing that this is a valid method to estimate MI area in rats.

### Hemodynamic assessments

Twenty-four hours after echocardiographic evaluation, 2 catheters filled with 0.06 mL of saline were implanted into the femoral artery and femoral vein of the anesthetized rats (80 mg/kg ketamine and 12 mg/kg xylazine, i.p.). On the next day, the arterial cannula was connected to a strain-gauge transducer (Blood Pressure XDCR; Kent Scientific, Torrington, CT), and arterial pressure (AP) signals and pulse intervals (PI) were recorded over a 30-minute period in conscious animals, as previously described [16,17].

Sequential bolus injections (0.1 mL) of increasing doses of phenylephrine (0.25-32 mg/kg) and sodium nitroprusside (0.05-1.6 mg/kg) were given to induce increases or decreases in mean AP responses (for each drug), ranging from 5 to 40 mmHg. Baroreflex sensitivity was expressed as bradycardic response (BR) and tachycardic response (TR) in beats per minute per millimeter of mercury, as described elsewhere [16,17].

### Cardiac autonomic modulation

The overall variability of the pulse interval (PI) was assessed in the time and frequency domains by spectral estimation in the 30-minute recorded basal period. Fluctuations in PI were further assessed in the frequency domain by means of autoregressive spectral estimation, as described elsewhere [29,30]. Briefly, the PI series derived from each recording were divided into 300 beat segments with a 50% overlap. The spectra of each segment were calculated via the Levinson-Durbin recursion and the order of the model chosen according to Akaike’s criterion, with the oscillatory components quantified in LF (0.2-0.6 Hz) and high frequency (HF; 0.6–3.0 Hz) ranges. The normalized units were obtained by calculating the power of LF and HF correlating each to the total power, after subtracting the power of the very LF component (frequencies < 0.2 Hz).

### Statistical analyses

Statistical analyses were performed with SPSS software (Version 17.0 for Windows; SPSS Inc., Chicago, USA). Data are reported as mean ± SEM. After confirming that all continuous variables were normally distributed using the Kolmogorov–Smirnov test, statistical differences between the groups in all evaluations were obtained by two-way (exercise training and myocardial infarction) analysis of variance (ANOVA) followed by the Bonferroni post-test. Statistical differences between the data from maximal treadmill exercise test measured at the beginning of the protocol (initial evaluation), after ET or following protocols (final evaluation), and 2 days after *Sham*/MI surgeries were assessed using repeated-measures ANOVA. Pearson’s correlation was used to study the association between variables. The survival analyses were estimated by the Kaplan–Meier method and compared by the log-rank (Mantel–Cox) test. All tests were two sided and the significance level was established at P < 0.05.

## Results

### Mortality evaluation

During exercise training protocol or after *Sham* surgery, no death was registered in experimental groups. After MI surgery, mortality rate was higher in I animals (3 deaths among 12 rats, 25 %) when compared to TI (no deaths).

### Animals

Body weight was similar among all studied groups at the beginning of the protocol (~289 ± 6 g). At the end of the protocol, all experimental groups increased body weight when compared to their initial values (C: 422 ± 5; TC: 368 ± 10; I: 410 ± 5; TI: 367 ± 10 g); however, TC and TI groups had reduced body weight when compared to C and I groups, respectively. Similarly, retroperitoneal adipose tissue weight was decreased in TC (2.4 ± 0.2 g) and TI (2.2 ± 0.2 g) rats when compared to C (5.2 ± 0.1 g) and I (4.9 ± 0.1 g) rats.

Maximal running speed values obtained at initial, final and after *Sham* or MI surgeries are presented in Table 1. At the initial evaluation, maximal running speed was similar between the groups; however, at the final evaluation TC and TI groups presented increased physical capacity when compared to C and I groups, as well as in relation to their initial evaluation. After *Sham* surgery, physical capacity remained similar in C and CT animals when compared to their final evaluations; however, after MI, I animals presented decreased physical capacity when compared to C animals, and to their initial and final evaluations. On the other hand, ET prevented an additional decrease in maximal running speed in TI rats (Table 1).

**Table 1:** Maximal running speed speed (Km/h) in sedentary control rats (C, n = 8); trained control rats (TC, n = 8); sedentary rats submitted to myocardial infarction (I, n = 9), trained rats submitted to myocardial infarction (TI, n = 10)

**Parameters/Groups**	**C**	**CT**	**I**	**TI**
Initial	1.20 ± 0.11	1.50 ± 0.06	1.20 ± 0.06	1.50 ± 0.06
Final	1.50 ± 0.06	2.40 ± 0.03#*	1.50 ± 0.03	2.40 ± 0.05#*†
Post-MI/*Sham*	1.50 ± 0.04	2.40 ± 0.05#*	0.90 ± 0.04#$*	1.20 ± 0.04$*†

Values are expressed as mean ± SEM. Repeated-measures ANOVA. #p < 0.05 vs. initial evaluation in the same group; $p < 0.05 vs. final evaluation in the same group; *p < 0.05 vs. C; †p < 0.05 vs. I.

### Left ventricular morphometry and function

The echocardiographic parameters of LV morphometry and function are shown in Table 2. MI area was similar between I and TI animals. Relative wall thickness was reduced in I group when compared to C group. LV mass and relative wall thickness values were increased in TC and TI groups when compared to C group. Furthermore, LV mass and relative wall thickness were also increased in TI animals when compared to I animals. Left ventricular end-diameter during diastole remained unchanged in experimental groups.

**Table 2:** Echocardiographic parameters in sedentary control rats (C, n = 8); trained control rats (TC, n = 8); sedentary rats submitted to myocardial infarction (I, n = 9), trained rats submitted to myocardial infarction (TI, n = 10)

**Parameters/Groups**	**C**	**TC**	**I**	**TI**
***Morphometric***				
MI area (%)	----	----	45 ± 3	42 ± 2
LVmass (g)	1.11 ± 0.03	1.62 ± 0.05*	1.14 ± 0.05	1.65 ± 0.11*†
LVDD (cm)	0.72 ± 0.02	0.74 ± 0.03	0.74 ± 0.02	0.76 ± 0.03
RWT	0.40 ± 0.01	0.53 ± 0.03*	0.29 ± 0.01*	0.51 ± 0.03*†
***Systolic Function***				
FS (%)	39 ± 1	41 ± 3	30 ± 2*	38 ± 1†
EF (%)	70.2 ± 0.9	76.1 ± 0.3*	41.2 ± 1.1*	52.4 ± 0.4*†
***Diastolic Function***				
E/A	1.61 ± 0.11	1.67 ± 0.01	2.76 ± 0.20*	1.81 ± 0.11†
IVRT (ms)	29 ± 1	27 ± 1	29 ± 1	28 ± 3
***Global Function***				
MPI	0.37 ± 0.03	0.15 ± 0.02*	0.48 ± 0.03*	0.18 ± 0.04*†

Values are expressed as mean ± SEM. LVmass - left ventricular mass; LVDD - left ventricular end-diameter during diastole; RWT – relative wall thickness; FS – fractional shortening; EF – ejection fraction; E/A – E wave A wave ratio; IVRT- left ventricular isovolumetric relaxation time; MPI – myocardial performance index. ANOVA two-way (exercise training and myocardial infarction) analysis followed by the Bonferroni post-test. *p < 0.05 vs. C; †p < 0.05 vs. I.

Regarding LV function, TC group improved ejection fraction and myocardial performance index when compared to C group. I rats demonstrated reduced systolic function parameters (fractional shortening and ejection fraction), as well as increased E/A ratio and myocardial performance index when compared to C rats. It should be stressed that ET was able to prevent systolic, diastolic and global dysfunction in TI animals, as observed by normalization of fractional shortening, E/A ratio and myocardial performance index. Although ET did not prevent low ejection fraction in TI group, these values remained higher when compared to the I group (Table 2).

### Hemodynamic evaluations

**Figure 1:**
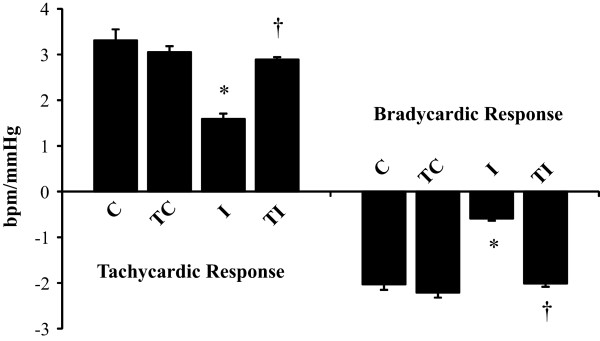
**Baroreflex sensitivity represented by tachycardic and bradycardic responses in sedentary control rats (C, n = 8); trained control rats (TC, n = 8); sedentary rats submitted to myocardial infarction (I, n = 9), trained rats submitted to myocardial infarction (TI, n = 10).** Values are expressed as mean ± SEM. ANOVA two-way (exercise training and myocardial infarction) analysis followed by the Bonferroni post-test. *p < 0.05 vs. C; †p < 0.05 vs. I.

Hemodynamic parameters can be observed in Table 3. No changes were observed in systolic, diastolic or mean arterial pressure between experimental groups. In contrast, heart rate was reduced in TC group when compared to C group. An increase in heart rate was observed in I animals when compared to C. This increase was prevented by previous ET, as observed in TI rats.Baroreflex sensitivity, evaluated by bradycardic and tachycardic responses evoked by arterial pressure rises and falls, was impaired in the I group when compared to C group (Figure 1). Previous ET was able to prevent the baroreflex sensitivity reduction, as observed in TI rats.

**Table 3:** Hemodynamic variables in sedentary control rats (C, n = 8); trained control rats (TC, n = 8); sedentary rats submitted to myocardial infarction (I, n = 9), trained rats submitted to myocardial infarction (TI, n = 10)

**Parameters/Groups**	**C**	**TC**	**I**	**TI**
SAP (mmHg)	122 ± 2	115 ± 3	115 ± 3	117 ± 4
DAP (mmHg)	86 ± 3	85 ± 2	80 ± 3	84 ± 2
MAP (mmHg)	98 ± 3	95 ± 2	92 ± 2	95 ± 4
HR (bpm)	319 ± 5	295 ± 4*	339 ± 9*	331 ± 4

Values are expressed as mean ± SEM. SAP – systolic arterial pressure; DAP – diastolic arterial pressure; MAP – mean arterial pressure; HR – heart rate. ANOVA two-way (exercise training and myocardial infarction) analysis followed by the Bonferroni post-test. *p < 0.05 vs. C.

### Cardiac autonomic modulation

**Figure 2:**
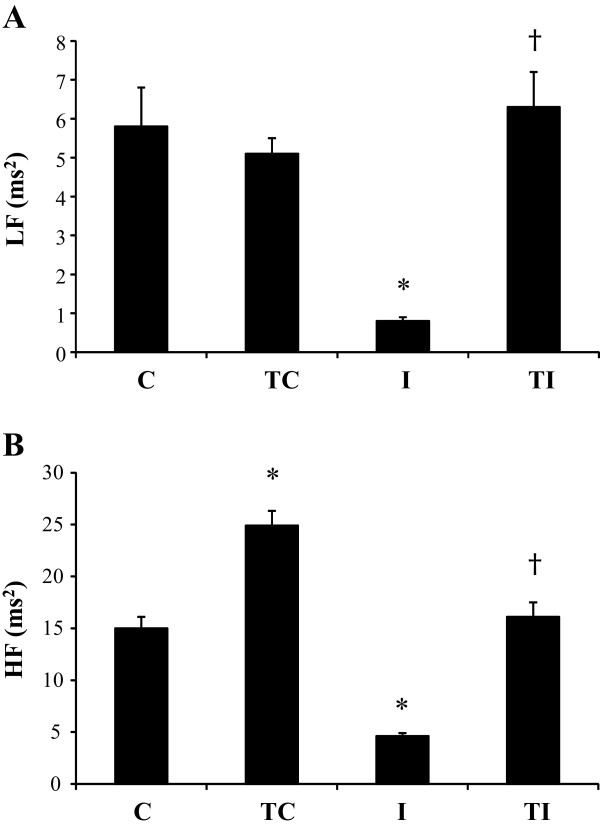
**Low frequency (indicative of sympathetic modulation, panel A) and high frequency (indicative of parasympathetic modulation, panel B) bands of pulse interval variability in sedentary control rats (C, n = 8); trained control rats (TC, n = 8); sedentary rats submitted to myocardial infarction (I, n = 9), trained rats submitted to myocardial infarction (TI, n = 10).** Values are expressed as mean ± SEM. ANOVA two-way (exercise training and myocardial infarction) analysis followed by the Bonferroni post-test. *p < 0.05 vs. C; †p < 0.05 vs. I.

Values of PI variability parameters are presented in Table 4. Impairments of Variance, RMSSD, absolute values of LF band (Figure 2A) and HF band (Figure 2B), as well as LF/HF of PI, were observed in I group when compared to C group. Previous ET was able to prevent the impairments of PI variability parameters in time and frequency domains, mainly related to absolute values of LF (Figure 2A) and HF (Figure 2B) bands, and autonomic balance, as observed in TI rats (Table 4).

**Table 4:** Pulse interval (PI) variability in time and frequency domains in sedentary control rats (C, n = 8); trained control rats (TC, n = 8); sedentary rats submitted to myocardial infarction (I, n = 9), trained rats submitted to myocardial infarction (TI, n = 10)

**Parameters/Groups**	**C**	**TC**	**I**	**TI**
SD (ms)	11.1 ± 0.8	10.1 ± 1.2	9.0 ± 0.8	10.7 ± 1.4
Variance (ms^2^)	116.5 ± 17.2	132.6 ± 25.9	23.2 ± 3.7*	107.8 ± 21.8†
RMSSD (ms^2^)	6.6 ± 0.2	6.7 ± 0.7	4.3 ± 0.2*	8.4 ± 0.6†
LF (%)	26.7 ± 2.6	20.0 ± 1.9	14.9 ± 1.4*	27.3 ± 3.3†
HF (%)	73.2 ± 2.7	82.8 ± 3.2	85.1 ± 1.4	72.6 ± 3.3
LF/HF	0.38 ± 0.05	0.26 ± 0.03	0.18 ± 0.02*	0.39 ± 0.06†

Values are expressed as mean ± SEM. SD - standard deviation of the PI variability; RMSSD - root-mean square of differences of successive RR intervals; LF – low frequency band; HF – high frequency band; LF/HF – autonomic balance. ANOVA two-way (exercise training and myocardial infarction) analysis followed by the Bonferroni post-test. *p < 0.05 vs. C; †p < 0.05 vs. I.

### Correlations

**Figure 3:**
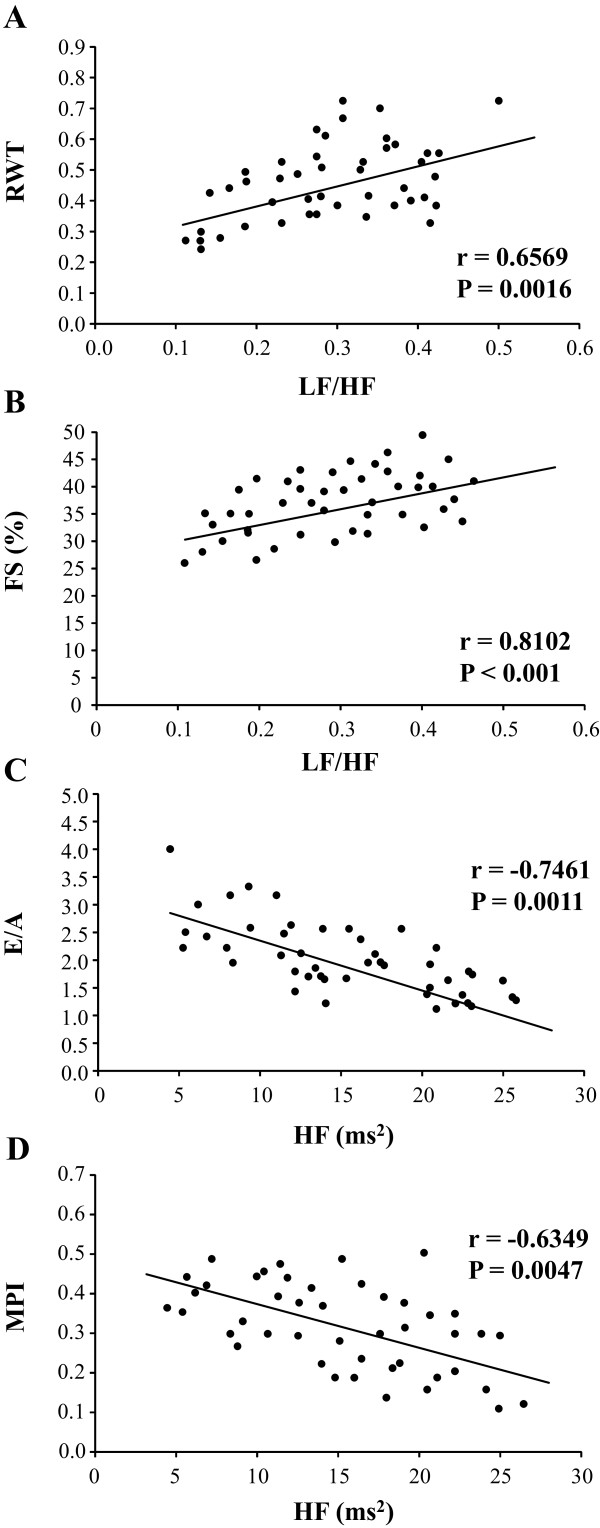
Pearson’s correlations between autonomic balance (LF/HF ratio) and: relative wall thickness (RWT, panel A) and fractional shortening (FS, panel B); as well as between high frequency band (HF) and: E/A ratio (panel C) and myocardial performance index (MPI, panel D).

Positive correlations were observed between LF/HF ratio and relative wall thickness (r = 0.6569; P = 0.0016) (Figure 3A), and with left ventricular fractional shortening (r = 0.8102; P < 0.001) (Figure 3B). On the other hand, negative correlations were obtained between absolute values of HF band and E/A ratio (r =-0.7461; P = 0.0011) (Figure 3C), and with myocardial performance index (r =-0.6349; P = 0.0047).

## Discussion

In recent years, pharmacological therapies and changes in lifestyle, including weight control, physical activity, smoking cessation and reduced consumption of calories in the diet, have contributed to the primary prevention of CVD. ET plays a key role in health promotion and has become a unanimous approach in cardiology [31-34]. The present work provides strong evidence for the attenuation of MI-induced cardiac dysfunction by prior to MI moderate-intensity ET, and, importantly, this effect was associated with the prevention of post-MI cardiac autonomic impairment. The cardioprotective effect of prior ET was also confirmed by the reduced peri-operative mortality observed in trained animals. To our knowledge, this is the first study to demonstrate that the attenuation of cardiac autonomic modulation changes may be an important mechanism associated with the cardioprotection conferred by ET.

Reduced exercise capacity after MI has been considered an important predictor of mortality [35,36]. In the present study, after inducing MI, maximum running speed was reduced in I group in relation to their initial and final evaluations, as well as when compared with C group. It is worth noting that ET has been effective in preventing a decline in aerobic capacity in previously trained rats undergoing MI. In line with our findings, Bozi et al. [23] have shown that the ET prior to MI also prevented further decline in physical capacity in rats, when evaluated by the total time of the exercise test. In addition, the efficacy of ET was also demonstrated by resting bradycardia in CT rats, and by preventing increases in heart rate in TI rats.

Left ventricular remodeling is an indicator of great impact on cardiac mortality after ischemia [37]; however, it is unclear how exercise affects this remodeling. Although no change in the MI area have been observed when infarction was induced following ET, left ventricular mass and relative wall thickness were further increased in TI animals. Although additional analysis of cardiomyocyte diameter and length was not carried out, and molecular markers of physiological and pathological hypertrophy were not researched, our data suggest that pathological cardiac remodeling was indeed prevented in the TI group, since systolic and diastolic dysfunction were attenuated by prior ET in these animals.

Likewise, Dayan et al. [18] have shown that three weeks of swimming training before MI attenuated ventricular remodeling and improved left ventricular function, despite unchanged cardiac dimensions. The authors have suggested that even a short period of training is sufficient to induce cardiac protection. In contrast, Veiga et al. [38] have not observed any differences on morphological parameters, cardiac function nor on the tension of the papillary muscles in swimming trained female rats prior to MI. It is possible that the observed differences between these findings and the results of the present study are due to the type of training performed (swimming vs. treadmill), as well as to the gender of the chosen animals (females vs. males). In fact, Bozi et al. [23] have demonstrated that ET on a treadmill attenuated cardiac dysfunction and structural deterioration promoted by MI. Similarly, 12 weeks of aerobic ET increased antioxidant enzymes, decreased oxidative damage and reduced the degree of MI induced by isoproterenol in male mice hearts [21]. Moreover, it has been shown that ET performed before MI reprograms the surviving myocardium and changes its molecular response, a fact that may account for, at least in part, cardioprotective phenotype of the exercised animals [39].

There are multiple mechanisms by which moderate to vigorous ET may decrease mortality rates associated with CVD, including antiatherosclerotic, antithrombotic, anti-ischemic, antiarrhythmic, and antioxidant effects [34,40]. In order to further investigate the possible candidate mechanisms associated with the attenuated cardiac dysfunction and exercise capacity in trained rats prior to MI, baroreflex sensitivity and cardiac autonomic modulation were investigated. In this context, since the classical studies of Billman et al. [41] and Hull et al. [42], there have been consistent findings pointing that ET reduces mortality in patients after MI, particularly when associated with increased vagal component and decreased sympathetic activity. In fact, most clinical and experimental studies indicate that improvement in baroreflex sensitivity and autonomic function as important ET adaptations after MI [11,13,14,16,17,43,44]; however, their cardioprotective role remains unclear.

It is well known that after MI catecholamine production and release from adrenal glands and from cardiac sympathetic nervous system nerve endings are enhanced [45]. In the failing heart, sympathetic activation results in changes in beta-adrenergic receptors [46,47] that plays a key role in left ventricular remodeling [48]. On the other hand, it has been reported that aerobic ET reduces and restores adrenal G protein-coupled receptor kinase-2 enzyme levels and activity, which results in marked reduction of adrenal catecholamine production [49]. In addition, 8 weeks of aerobic ET inhibits cardiac sympathetic nerve sprouting and restores β3-/β1 adrenoreceptors balance and increases the expression of β3 adrenoreceptor after MI, resulting in improvement of cardiac function [50].

In the present investigation, we demonstrated that moderate ET, when performed prior to MI, prevented baroreflex sensitivity impairment, sympathetic modulation increase and parasympathetic modulation decrease, as observed in TI rats. Furthermore, improvements in autonomic balance (represented by LF/HF ratio), and in parasympathetic modulation (HF band) were strongly correlated with structural, systolic, diastolic and global left ventricle function. Since the later development of left ventricular dysfunction has been associated with further increment in neurohumoral excitation, due to arterial and cardiopulmonary baroreceptors [51,52], our results indicate that autonomic parameters preservation by ET may be associated with attenuated cardiac function abnormalities in TI animals.

This work has some limitations that deserve comments. Although our group have previously showed strong correlations between the MI area assessed by echocardiogram and post mortem histological analysis [14,16,17,29], the lack of histological data is a limitation of the present study. In addition, although we have not performed biochemical analysis for markers of hypertrophy, remodeling and beta-adrenergic receptor signaling, we did a screening of cardiovascular and autonomic analyzes, providing important data related to the cardioprotection mechanisms afforded by ET.

## Conclusion

In summary, we have demonstrated that prior exercise training was effective in aerobic capacity, left ventricular morphology and function in rats undergoing myocardial infarction. Furthermore, these cardioprotective effects were associated with attenuated cardiac autonomic dysfunction observed in trained rats. Although these cause-effect relationships can only be inferred, rather than confirmed, our study suggests that positive adaptations of autonomic function by exercise training can play a vital role in preventing changes associated with cardiovascular disease, particularly in relation to MI. However, other systemic and local mechanisms cannot be ruled out. Thus, these findings encourage enhancing baroreflex sensitivity and cardiac autonomic function as a therapeutic strategy for the prevention of cardiac abnormalities triggered by myocardial ischemia.

## Abbreviations

CVD: Cardiovascular Disease; MI: Myocardial Infarction; ET: Exercise Training; C: Sedentary Control Rats Submitted To Sham Surgery; TC: Trained Control Rats Submitted To Sham Surgery; I: Sedentary Rats Submitted To Myocardial Infarction Surgery; TI: Trained Rats Submitted To Myocardial Infarction Surgery; LV: Left Ventricle; LVDD: Left Ventricular End-Diameter During Diastole; RWT: Relative Wall Thickness; FS: Fractional Shortening; EF: Ejection Fraction; E/A: E Wave A Wave Ratio; IVRT: Left Ventricular Isovolumetric Relaxation Time; MPI: Myocardial Performance Index; AP: Arterial Pressure; SAP: Systolic Arterial Pressure; DAP: Diastolic Arterial Pressure; MAP: Mean Arterial Pressure; HR: Heart Rate; PI: Pulse Interval; BR: Bradycardic Response; TR: Tachycardic Response; SD: Standard Deviation Of The Pulse Interval Variability; RMSSD: Root-Mean Square Of Differences Of Successive Rr Intervals; LF: Low Frequency Band; HF: High Frequency Band; LF/HF: Autonomic Balance.

## Competing interest

The authors have no conflicts of interest to declare.

## Authors’ contributions

FR: Data acquisition, analysis and interpretation; DJF: Data acquisition, analysis, interpretation; CAB: Data acquisition and analyze; MEVA: Data acquisition and analysis; LYR: Data acquisition and analysis; NMC: Data acquisition; CM: Data analysis and interpretation; DF: Data acquisition and analyze; GIHS: Data analysis and interpretation; KDA: Statistical analysis and helped to draft the manuscript; MCI: interpretation of data and helped to draft the manuscript; BR: conception and design of the work and draft the manuscript. All authors approved final version to be published.

## Pre-publication history

The pre-publication history for this paper can be accessed here:

http://www.biomedcentral.com/1471-2261/14/84/prepub
